# Ophthalmic surgeries before and during the covid-19 outbreak in a tertiary hospital

**DOI:** 10.1007/s10792-022-02555-4

**Published:** 2022-10-15

**Authors:** Yasemin Fatma Cetinkaya

**Affiliations:** Department of Ophthalmology, Gulhane Training and Research Hospital, Ankara, Turkey

**Keywords:** COVID-19, Ocular surgery, Healthcare, Eye health, Pandemic

## Abstract

**Objective:**

As with any healthcare practice, elective surgeries had to be postponed since the start of the Covid-19 pandemic. This study aimed to examine the characteristics of ophthalmology outpatients and eye surgery admissions during the COVID-19 pandemic and also to compare the pandemic and pre-pandemic periods.

**Methods:**

This retrospective study included patients admitted to the ophthalmology clinic of a tertiary hospital from April through June 2020. A control sample was formed using the registries from the same interval in the previous year. The primary endpoint was the difference between the number and distribution of types of surgical procedures in the pre-pandemic and pandemic period. Surgical procedures were classified as Group A; major special, Group B; special, Group C; major, Group D; medium, and Group E; minor surgeries. Also surgeries were also divided into 4 groups. Cataract and related surgeries were grouped as “Phaco”, emergency surgeries for trauma patients as “Trauma”, retina and related surgeries were grouped as “Retina”, and eyelid and adnexal surgeries were grouped as “Eyelid”. The secondary endpoint was the comparison between the pre-pandemic and pandemic period.

**Results:**

A total of 116 operations were performed in 2020 (mean age: 42.3 ± 25.6 years, male: 63.8%). In 2019, 873 surgeries were performed in the same period of the year (mean age: 56.6 ± 20.2 years, male: 48.8%), indicating an 86.7% decrease during the pandemic period, and each surgery type reduced significantly. On the other hand, the proportion of Group A (10.3% in 2019 vs. 25.9% in 2020, *p* < 0.001), group B (5.4% in 2019–17.24% in 2020, *p* < 0.001), and group E (3.8% in 2019–8.6% in 2020, *p* < 0.001) surgeries among the total increased in the pandemic period. The proportion of trauma (3.1% in 2019–16.4% in 2020, *p* < 0.001) and retina (11.9% in 2019–37.1% in 2020, *p* < 0.001) surgeries also increased, whereas phaco and eyelid surgeries were recorded at a lesser rate in the pandemic period.

**Conclusion:**

This study showed a striking reduction in eye surgery during the early period of the Covid-19 pandemic. However, the rates of group A, B, and E surgeries increased significantly compared to the previous year.

## Introduction

The primary mode of transmission among SARS-CoV-2 individuals is direct respiratory or droplet transmission, but infection can also be transmitted through the nose, eyes, and mouth following contact with the surfaces contaminated with the virus [1]. SARS-CoV-2 has also been detected in non-respiratory samples, including stool, blood, ocular secretions, and semen, although the role of these sites in the transmission is unclear [2]. Recent research has described the biology, transmission, and diagnosis of COVID-19, its clinical features, and the progress of vaccines and therapeutics; however, more data on the impact of COVID-19 on the national health systems are needed [3]. In this context, the impact of the pandemic on access to eye care and salvage treatments for vision, which can cause disability for many patients, needs to be explored. It is also essential to determine the potential risks, reduce unnecessary outpatient visits, and perform elective surgeries timely, especially in elderly patients and patients with comorbidities. This study examined how routine eye surgery practice has been influenced during a global pandemic. In this way, awareness can be increased to take precautions and avoid excessive costs.

Turkey has taken necessary measures across the country from April 2020 to prevent the transmission of SARS-CoV-2. A curfew was introduced, restricting all outdoor movements except essential services. Regions were managed with local measures in line with the decisions of the provincial sanitation boards according to the number of COVID cases [4]. In line with the measures taken, elective surgeries were postponed not only to protect the patients and healthcare staff but also to meet the increasing need for beds. Therefore, the number of surgeries was reduced, reserving the limited space for emergency cases.

Our aim in this study was to investigate how eye surgery practice was affected by the COVID-19 pandemic, examine the changes in the distribution of eye surgery priorities, and compare the findings with the pre-pandemic period.

### Subject and method

We conducted a retrospective study using outpatient and inpatient admissions at the Gülhane Training and Research Hospital, Ankara, Turkey. We determined the total eye surgeries performed in April, May, and June in pre-pandemic 2019 and compared them with the number of surgeries in the same months in the pandemic year 2020. Patients who were aged 18 years and older were admitted either for an emergency or as an outpatient. Because the curfew was strictly enforced and families avoided bringing their children to the hospital, admissions under the age of 18 were very few and they were excluded from the study. No other exclusion criteria were applied. The study was approved by the institutional review board (08/07/2021, Code: 46,418,926), and the study design conforms to the principles outlined in the revised version of the Declaration of Helsinki.

### Data collection

The data were obtained using the continuous health records register of a tertiary care center. Demographic data, number of surgeries, types of surgeries, and length of hospital stay were recorded. Admissions to the retina, cornea, lens, oculoplasty, strabismus, glaucoma, ocular oncology, low vision aids, neuro-ophthalmology, contact lenses, and pediatric ophthalmology units were recorded.

Surgeries were classified into 4 groups. Cataract and related surgeries were grouped as “Phaco”, emergency surgeries for trauma patients as “Trauma”, retina and related surgeries were grouped as “Retina”, and eyelid and adnexal surgeries were grouped as “Eyelid”. In addition, five groups (A, B, C, D, and E) were formed based on the size of the surgery using the scoring system endorsed by the Turkish Ministry of Health. (Group A: major special surgeries and interventions, Group B: special surgeries and interventions, Group C: major surgeries and interventions, Group D: medium surgeries and interventions, and Group E: minor surgeries and interventions).

The length of hospital stay was the period between the day of hospitalization of the patient in the emergency service or the ophthalmology clinic and the day of postoperative discharge.

### Statistical analysis

R Studio (Version 3.6.2) was used for statistical analysis. Continuous variables were given as mean ± standard deviation (SD). Two proportions of the Z-test were used to compare surgery proportions. *P* < 0.05 was considered statistically significant.

## Results

The number of surgeries performed in 2020 was 116 (age (mean ± SD): 42.3 ± 25.6 years, male: 63.8%), whereas 873 surgeries (age (mean ± SD): 56.6 ± 20.2 years, female: 48.8%) were performed in 2019 (Table 1). The reduction in the number of surgeries was 86.7% in the first three months of the Covid-19 pandemic. Male predominance was observed in the pandemic period, while the gender distribution was similar in 2019. Also, the mean age of the patients was markedly younger during the pandemic period.

**Table 1:** Basic characteristics

	Pre-pandemic year (2019) (*n* = 873)	Pandemic year (2020) (*n* = 116)
Age, years, mean ± SD	56.6 ± 20.2	42.3 ± 25.6
Male, n (%)	426 (48.8)	74 (63.8)
Female, n (%)	447 (51.2)	42 (36.2)
Length of hospitalization, mean ± SD	3.95 ± 11.9	9.87 ± 23.6

Phaco was the most common type of surgery in both 2019 (*n* = 357) and 2020 (*n* = 15). On the other hand, Phaco surgeries decreased markedly from 2019 to 2020 (*p* < 0.001). The number of trauma operations was 27 and 19 in 2019 and 2020, respectively. Although the number of trauma surgeries was lower in 2020, its overall proportion among all surgeries increased significantly in 2020 (*p* < 0.001). Retina surgeries also decreased from 100 in 2019 to 43 in 2020. Similar to the trauma surgeries, despite lower numbers recorded in 2020, the proportion of retina surgeries increased from 11.9% in 2019 to 37.1% in 2020 among the total number of surgeries. The number of eyelid surgeries reduced from 117 in 2020 to 1 in 2020 (*p* < 0.001) (Table 2). While 19 strabismus surgeries were performed in 2019, no record was found in 2020.

**Table 2:** Types of operations in the pre-pandemic and pandemic periods

	Pre-pandemic year (2019)	Pandemic year (2020)	Z-statistics	*p*-value
Phaco, n (%)	357 (40.9)	15 (12.9)	5.739	< 0.001
Trauma, n (%)	27 (3.1)	19 (16.4)	−6.150	< 0.001
Retina, n (%)	100 (11. 9)	43 (37.1)	−7.229	< 0.001
Eyelid, n (%)	127 (14.6)	1 (0.9)	3.978	< 0.001

### Evaluation of the surgery classifications and length of hospital stay

The number of operations across surgery groups also showed differences in the pandemic period compared with the pre-pandemic period (Table 3). The number of group A, B, and E surgeries increased significantly in 2020. However, there was a significant decrease in the number and percentage of group C and D surgeries compared with the same period in 2019 (Table 3). Contrary to the expectations, the duration of hospitalization during the pandemic period increased significantly from 3.95 ± 11.9 days to 9.87 ± 23.6 days.

**Table 3:** Groups of operations in the pre-pandemic and pandemic periods

Groups	Pre-pandemic year (2019)	Pandemic year (2020)	Z-Statistics	p-value
A, n (%)	90 (10.3)	30 (25.9)	−4.669	< 0.001
B, n (%)	47 (5.4)	20 (17.2)	−4.578	< 0.001
C, n (%)	436 (49.9)	36 (31.0)	3.732	< 0.001
D, n (%)	267 (30.6)	20 (17.2)	2.866	0.004
E, n (%)	33 (3.8)	10 (8.6)	−2.160	0.031
Total	873 (100)	116 (100)		

## Discussion

This study showed a striking reduction in the number of eye surgeries during the early period of the Covid-19 pandemic. However, an increase in trauma-related surgeries was found observed. Our aim in this study was to investigate how the surgical ophthalmology practice was affected by the COVID-19 pandemic. The data obtained in the current study may be useful in planning more effective health care delivery.

Phaco was the most common type of surgery in both 2019 and 2020 in our analysis. On the other hand, the number of Phaco surgeries decreased significantly from 2019 to 2020 (*p* < 0.001). The number of trauma surgeries was also lower in 2020; however, its overall proportion among all surgeries increased significantly in 2020. Surgeries due to trauma (immediate non-deferrable surgeries) were particularly related to firearm injuries during the pandemic. This may be caused by the long-standing role of our hospital as a referral center for the wounded military staff. Retina surgeries also decreased from 2019 to 2020. Similar to the trauma surgeries, despite lower numbers recorded in 2020, the proportion of retina surgeries increased more than threefold in 2020 among the total number of surgeries. The number of eyelid and strabismus surgeries markedly reduced from 2019 to 2020.

Compared to the pre-pandemic period, the groups of surgeries differed during the pandemic period (Table 3). Although the total number of groups A, B, and E surgeries decreased in 2020, their proportion in the total number of operations was higher than the previous year. This could be explained by the fact that group A and B surgeries mostly involve trauma and retinal surgeries. In addition, the ratio in the total number remained stable, as there were minor interventions (such as removal of the foreign body in the eye) in group E. The low number of operations in groups C and D could be explained by the fact that these groups mainly included elective interventions such as phaco, strabismus, or eyelid surgery. As expected, this situation was found to be compatible with the other surgery group rates (Table 2).

The pandemic and quarantine have created a wide range of challenges for healthcare services. While restrictions and worries of getting infected by the virus concerned the patients and overall population, even more distress influenced the healthcare professionals, limiting the routine outpatient and inpatient practice.

A recent report by the CovidSurg Collaborative predicted that 28.4 million surgeries worldwide were canceled or postponed in 2020 [9]. Elective surgeries have also decreased significantly, including the eye units. On the other hand, reviewing all scheduled procedures should not necessarily entail canceling all elective surgery cases. Given the uncertainty about how long COVID-19 will last, excessive cancelations might have a more dramatic and immeasurable impact on public health than the morbidity and mortality caused by the Covid-19. Recent studies also examined the impact of the pandemic on ocular trauma and emergency trauma cases. An online survey showed that most ophthalmologists did not examine patients and that elective surgeries were almost completely postponed during the early stages of the pandemic [5, 6]. Other studies from different countries have evaluated the impact of quarantine on healthcare and especially ophthalmic services and displayed similar results [7, 8]. Our findings are overall in agreement with data obtained in other regions of the world.

Our study recorded male predominance and younger age of the patients operated during quarantine. This may be due to the stronger quarantine measures announced for older adults who had to stay home for longer periods. The current observation is consistent with a previous report from a tertiary hospital setting during the lockdown [10].

Surgical procedures require a significant allocation of equipment and resources, not only in the operating room but also in postoperative care units. Therefore, the need for reserving the workforce for emergency procedures was reasonable in the earlier periods of the pandemic. However, several algorithms were quickly announced by the national surgical societies for triage in eye care during the resource shortage. Nevertheless, many non-urgent elective eye surgeries become urgent at some point during the pandemic.

We did observe a lower number of trauma-related eye surgeries during the lockdown, similar to a previous study that showed a 41.8% reduction in mechanical trauma cases at an eye unit during quarantine [11]. As there was a more significant reduction in the total surgeries during the lockdown, there was a relative increase in the proportion of trauma cases among all surgeries in 2020. The trauma-related surgeries (operations that cannot be immediately postponed) were particularly related to gunshot wounds. Such patients are primarily referred to our center, a specialized unit for the care of injured military staff.

Retinal surgeries also decreased in 2020. On the other hand, similar to trauma surgeries, the proportion of retinal surgeries among the total number of surgeries increased during the lockdown. Retina surgery is more common among middle-aged and older adults, who had stayed home over one year after the pandemic had been announced. The reduction in the number of eyelid surgery and strabismus surgery is predictable as both procedures could have been postponed by the patients themselves due to their lesser consequences on daily life.

Contrary to the expectations, the length of hospital stay during the pandemic period increased more than twofold. This may be caused by the fact that, as mentioned earlier, the number of surgeries due to trauma and retina disease increased during the pandemic, while phaco surgeries reduced. The former two surgeries require a longer inpatient observation, whereas the latter is mostly a one-day intervention.

The pandemic and the ensuing lockdown have certainly impacted disease prognosis, healthcare delivery, and resource pooling. Understanding the changing trends will help to be more prepared if a similar cycle is to be repeated in the future. It is necessary to develop long-term surgical strategies to increase the safety of elective surgeries such as cataracts and refractive surgery, which should be performed during the pandemic period. It should be questioned whether all outpatients should be tested preoperatively, especially if faster tests are present [12]. Another practice change that may decrease patient exposure is to make bilateral cataract surgery if possible [15]. This is not the chosen practice, but it could facilitate treatment while decreasing the risk during the COVID-19 pandemic.

The protection of healthcare personnel is also extremely important. New methods should be developed in both respects. As an example of the methods developed for this purpose; Aman Chandra, MD, University Hospital Southend, UK, shared a new idea of forming a microscope shroud to decrease aerosol transmission during intraocular surgery [18]. From Iran, Peyman et al. have developed and rapidly adapted a shield in biomicroscopes to protect patients and the ophthalmologist [19]. We need many more changes like this in our new normal, the pandemic. An important contribution to pandemic medicine was to raise awareness about telemedicine [13]. Telemedicine had already progressed to a great extent in recent years. The COVID-19 pandemic has accelerated the legal adoption of telehealth practices in many countries, at least temporarily, helping to further increase its availability [14–19].

Several limitations of the current study should be acknowledged. The study was performed at a facility that is a continuum of a former military hospital and receives admissions from dispatched military casualties, suggesting lesser generalizability of the findings to other units. Moreover, unlike any tertiary hospital, admission of referral casualties at different times of the pandemic continued uninterrupted, independent of the pandemic. Secondly, the study covers the first three months of the lockdown. Although the screened period represents the most complex lockdown period and presents the results in the worst scenario, a broader picture of a longer period following adaptations to the quarantine measures could also be worthwhile. Finally, as the study design was cross-sectional, the results do not provide information about the longitudinal changes in the number and proportion of surgery types in the relaxed periods of the pandemic.

In conclusion, this study examined how routine eye surgery practice has been influenced during a global pandemic. The findings emphasize that, under pandemic conditions, the delay of elective ophthalmologic surgeries was inevitable, and the number of patients admitted to eye surgery units decreased significantly. It seems imperative to design and implement algorithms focused on community health and patient safety, for both appropriate eye care provision and improved decision-making processes.

## References

[1] Cevik M, Marcus JL, Buckee C, Smith TC (2020). Severe acute respiratory syndrome coronavirus 2 (SARS-CoV-2) transmission dynamics should inform policy. Clin Infect Dis.

[2] Zhou P. A pneumonia outbreak associated with a new coronavirus of probable bat origin. caused used *Nature.* 2020 579: 270–273 10.1038/s41586-020-2012-7 PMC7095418 32015507

[3] WHO. 2020. Coronavirus Disease (COVID-19) Pandemic

[4] Naharci MI, Katipoglu B, Tasci I (2020). Coronavirus 2019 (COVID-19) outbreak and geropsychiatric care for older adults: a view from Turkey. Int Psychogeriatr.

[5] Madanagopalan VG, Gopal MS, Sengupta S (2020). Perspectives of physicians in general and ophthalmologists in particular about restarting services post-COVID-19 lockdown. Indian J Ophthalmol.

[6] Agarwal R, Sharma N (2020). Commentary: COVID-19 pandemic and national lockdown: The cascading effect. Indian J Ophthalmol.

[7] Sharma N, D'Souza S, Nathawat R, Sinha R, Gokhale NS, Fogla R (2020). All India Ophthalmological Society - Eye Bank Association of India consensus statement on guidelines for cornea and eye banking during COVID-19 era. Indian J Ophthalmol.

[8] Hamroush A, Qureshi M, Shah S (2020). Increased risk of ocular injury seen during lockdown due to COVID-19. Cont Lens Anter Eye.

[9] COVIDSurg Collaborative.Mortality and pulmonary complications in patients undergoing surgery with perioperative SARS-CoV-2 infection: an international cohort study *Lancet* 2020 396: 27-38gery during the COVID-19 10.1016/S0140-6736(20)31182-X PMC7259900 32479829

[10] Khanna RC, Honavar SG, Metla AL, Bhattacharya A, Maulik PK (2020). Psychological impact of COVID-19 on ophthalmologists-in-training and practicing ophthalmologists in India. Indian J Ophthalmol.

[11] Mishra D, Nair AG, Gandhi RA, Gogate PJ, Mathur S, Bhushan P (2020). The impact of COVID-19 related lockdown on ophthalmology training programs in India – Outcomes of a survey. Indian J Ophthalmol.

[12] Reddy JC, Vaddavalli PK, Sharma N, Sachdev MS, Rajashekar YL, Sinha R (2020). A new normal with cataract surgery during COVID-19 pandemic. Indian J Ophthalmol.

[13] Jayadev C, Mahendradas P, Vinekar A, Kemmanu V, Gupta R, Pradhan ZS (2020). Tele-consultations in the wake of COVID-19 – Suggested guidelines for clinical ophthalmology. Indian J Ophthalmol.

[14] Parikh D, Armstrong G, Liou V, Husain D (2020). Advances in Telemedicine in Ophthalmology. Semin Ophthalmol.

[15] Kohnen TJ (2020). The new normal for cataract and refractive surgery due to COVID-19 (SARS-CoV-2). Cataract Refract Surg.

[16] Clinical education. *ASCRS*. Available at: https://ascrs.org/clinical-education. Accessed, 2020

[17] Peyman A, Pourazizi M (2020). COVID-19 and ophthalmologists: introducing a simple protective shield for slitlamp biomicroscopic examination. J Cataract Refract Surg.

[18] ESCRS Player. Available at: https://player.escrs.org/. Accessed April 28, 2020.

[19] Implementing teleophthalmology during covid-19 pandemic. *ASCRS*. Available at:https://ascrs.org/clinical-education/webinars/2020-webinar-implementing-tele-ophthalmology-during-covid-19-pandemic. Accessed April 28, 2020.

